# Differential expression of miRNAs and functional role of mir-200a in high and low productivity CHO cells expressing an Fc fusion protein

**DOI:** 10.1007/s10529-021-03153-7

**Published:** 2021-06-16

**Authors:** Laura Bryan, Michael Henry, Niall Barron, Clair Gallagher, Ronan M. Kelly, Christopher C. Frye, Matthew D. Osborne, Martin Clynes, Paula Meleady

**Affiliations:** 1 National Institute for Cellular Biotechnology, Dublin City University, Glasnevin, Dublin 9, Ireland; 2 National Institute for Bioprocessing Research and Training, Dublin 4, Ireland; 3 School of Chemical and Bioprocess Engineering, University College Dublin, Belfield, Dublin 4, Ireland; 4 Eli Lilly and Company, LTC-North, 1200 Kentucky Avenue, Indianapolis, IN 46225 USA; 5 Eli Lilly, Kinsale Limited, Cork, Ireland

**Keywords:** Chinese hamster ovary (CHO) cells, MiRNA, Label free quantitative proteomics, Cell specific productivity (Qp), Biopharmaceuticals

## Abstract

**Objectives:**

We used miRNA and proteomic profiling to understand intracellular pathways that contribute to high and low specific productivity (Qp) phenotypes in CHO clonally derived cell lines (CDCLs) from the same cell line generation project.

**Results:**

Differentially expressed (DE) miRNAs were identified which are predicted to target several proteins associated with protein folding. MiR-200a was found to have a number of predicted targets associated with the unfolded protein response (UPR) which were shown to have decreased expression in high Qp CDCLs and have no detected change at the mRNA level. MiR-200a overexpression in a CHO CDCL was found to increase recombinant protein titer by 1.2 fold and Qp by 1.8 fold.

**Conclusion:**

These results may suggest a role for miR-200a in post-transcriptional regulation of the UPR, presenting miR-200a as a potential target for engineering industrially attractive CHO cell phenotypes.

**Supplementary Information:**

The online version contains supplementary material available at 10.1007/s10529-021-03153-7.

## Introduction

The Chinese hamster ovary (CHO) cell is widely accepted as the cell line of choice for the production of large scale commercial therapeutic proteins (Walsh 2018). The widespread use of these cells in therapeutic protein production can in part be attributed to their high productivity, human-like post-translational modifications and stable transgene expression (Kim et al. 2012). Most industrial advancements in CHO cell production capabilities to date can be attributed to optimised feeding strategies and adaption to serum-free medium (Kim et al. 2012). However, cell engineering strategies provide the opportunity to generate multiple CHO cell phenotypes important to the bioprocess.

Molecular profiling of CHO cells with diverse phenotypes can contribute to a greater understanding of the biology underpinning these phenotypes. The CHO cell genome was first sequenced in 2011 (Xu et al. 2011). This has provided the data required to investigate the genome of CHO cells as it relates to CHO cell metabolic pathways. Also in recent years data relating to the proteome, phosphoproteome, glycoproteome and miRnome have become available by the collaborative efforts of many researchers (Hackl et al. 2011; Becker et al. 2011; Baycin-Hizal et al. 2012; Henry et al. 2017; Yang et al. 2018). The information generated by these studies has been key to advancing the fundamental knowledge of CHO cell biology.

MiRNA targeted cell engineering strategies are of considerable interest due to their potential to alter entire pathways in attempts to improve bioprocess phenotypes, without interfering with the translational machinery of the cell (Hackl et al. 2012). MiRNAs are non-coding, which allows the cell’s processing machinery to focus on protein production (Bartel 2009). MiRNAs are crucial for the normal animal development and have also been shown to be involved in a number of biological processes (Fu et al. 2013). MiRNAs are also highly conserved across a vast range of species (Brennecke et al. 2005; Xie et al. 2005; Krek et al. 2005). Each miRNA targets a gene or genes by negatively regulating them at the post-transcriptional level (Ambros 2003). MiRNA engineering presents a powerful tool for the generation of industrially desirable CHO cell phenotypes. Previously Barron et al. applied miRNA engineering to generate industrially beneficial phenotypic changes in CHO cells with the over-expression of miR-7 in CHO-SEAP cells to target the growth and productivity phenotype (Barron et al. 2011).

In this study, we selected six recombinant CHO clonally derived cell lines (CDCLs) from a fed-batch shake flask study, which exhibited varying levels of productivity of an Fc-fusion protein, for comparison. CDCLs were grouped as high or low producing and compared at day 6 of culture. Both quantitative LC–MS/MS proteomic analysis and MiRNA profiling was carried out on high and low Qp Fc fusion protein producing CHO CDCLs. These datasets were then combined with mRNA information from an earlier study by Clarke and colleagues (2019) to form a multi-omic approach to characterise the intracellular pathways associated with the high and low Qp phenotypes in industrially relevant CHO CDCLs. Expression of these targeted miRNAs could then be manipulated to modulate the growth and/or productivity of Fc-fusion producing CHO CDCLs. A number of DE miRNAs were identified which were found to target proteins associated with protein folding. MiR-200a was found to have increased expression in high Qp CDCLs, and several predicted targets of this miRNA with decreased expression in high Qp CDCLs were identified. Functional investigation revealed that overexpression of miR-200a in CHO cells resulted in a reduction in VCD and an increase in titer and Qp, presenting miR-200a as a potential target for cellular engineering of CHO cell CDCLs with optimised protein production.

## Materials and methods

### Fed-batch cultivation of CHO CDCLs

CDCLs were seeded in E250 mL shake flasks containing 100 mL of Lilly propriety production medium at 0.75 × 10^6^ cells/mL. CDCLs were cultured in duplicate at 150 rpm, 6% CO_2_ and 35 °C for 14 days in an ISF1-XC Climo-shaker (Kühner). Cell density and viability were determined using an automated Vicell™ XR cell viability analyzer (Beckman Coulter, Brea, CA). Cell specific productivity was measured using the following calculation as previously described (Clarke et al. 2011).

1 $${\text{Qp}}\left( {{\text{pg}}/{\text{cell}}/{\text{day}}} \right) = {\text{~}}\left[ {\frac{{{\text{titre}}2 - {\text{titre}}1}}{{\left( {{\text{density}}2 - {\text{density}}1} \right)}}} \right] \times {\text{~daily~growth~rate}}$$

where

$$Daily\,growth\,rate = \frac{{(\ln \left( {density2} \right) - \ln \left( {density1} \right))/\left( {time2 - time1} \right)}}{{24}}$$

### Protein A HPLC

Analytical protein A chromatography was used to determine protein concentration (titer). An Applied Biosystems POROS A 20 μm, 2.1 × 30 mm column (Applied Biosystems, Foster City, CA) with an Agilent 1100 high performance liquid chromatography (HPLC) with UV detection (Agilent Technologies, Santa Clara, CA) was used for protein A chromatography.

### Protein extraction and in-solution protein digestion

Duplicate flasks of 3 high Qp and 3 low Qp CDCLs were grown in a 14-day fed batch shake flask study as described above and cell pellets were taken on day 6 of culture. Cell pellets were harvested by centrifugation at 1000×*g* for 5 min. Pellets were then washed in phosphate buffered saline and supernatant was removed. Pellets were stored at − 80 °C until cell lysis was performed. Approximately 2 × 10^6^ cells (corresponding to 1 mg protein) were lysed using 1 mL of lysis buffer, and then centrifuged at 14,000×*g* for 15 min. 1 μL of 0.5 M DDT was added to the lysate. The lysate was heated for 20 min at 56 °C using a heating block to denature the protein. Protein concentration was determined using a Bradford assay (Bio-rad) as per manufacturer’s instructions and 100 μg of each sample was prepared for LC–MS/MS analysis. Samples were prepared using the Filter Aided Sample Preparation (FASP) method and C18 peptide purification as described previously (Coleman et al. 2017).

### LC–MS/MS

Reverse-phased capillary high pressure liquid chromatography (HPLC) was used for profiling total protein lysates of high and low Qp CHO CDCLs. This was performed on an UltiMate 3000 nano RSLC (Thermo Scientific) system in combination with an Orbitrap Fusion Tribrid Mass Spectrometer (MS) (Thermo Scientific). The equivalent of 1 µg of peptide was loaded onto the trapping column (PepMap100, C18, 300 µm × 5 mm) at a flow rate of 25 µL/min with 2% (v/v) acetonitrile (ACN), 0.1% (v/v) trifluoroacetic acid (TFA) for 3 min. The sample was then resolved onto an analytical column (Acclaim PepMap 100, 75 µm × 50 cm, 3 µm bead diameter column). The binary gradient used to elute peptides was: solvent A (0.1% (v/v) formic acid in LC–MS grade water) and solvent B (80% (v/v) ACN, 0.08% (v/v) formic acid in LC–MS grade water) using 2–32% B for 75 min, 32–90% B in 5 min and holding at 90% for 5 min at a flow rate of 300 nL/min. A voltage of 2.0 kV and a temperature of 320 °C was used for peptide ionization. A full scan range of 380–1500 m/z was used for data-dependent acquisition. Scans were performed using the Orbitrap mass analyser with a resolution of 120,000 (at m/z 200), a maximum injection time of 50 × ms and an automatic gain control (AGC) value of 4 × 10^5^. The top-speed acquisition algorithm was used to determine the number of selected precursor ions for fragmentation. An isolation width of 1.6 × Da was used to isolate selected precursor ions in the Quadrupole. A dynamic exclusion was applied to the analysed peptides after 60 s and only peptides with a charge state between 2 + and 7 + were analysed. Higher energy collision-induced dissociation with a normalized collision energy of 28% were used for fragmenting precursor ions, and the resulting MS/MS ions were measured in the linear ion trap. MS/MS scan conditions were typically the following: a targeted AGC value of 2 × 10^4^ and a maximum fill time of 35 ms.

### LC–MS/MS analysis

Proteins were identified using Proteome Discover version 2.1 software (Thermo Scientific) using the SEQUEST HT algorithm and the Uniprot CHO database (downloaded June 2019 containing 23,968 sequences). The following criteria was applied to all Proteome Discover searches: (1) precursor mass tolerance set at 20 ppm, (2) fragment mass tolerance set at 0.6 Da, (3) oxidation of methionine set as a dynamic modification, (4) carbamidomethylation of cysteine set as a static modification, and (5) a maximum of two missed cleavage sites was tolerated. Percolator was used to apply false-discovery rates (FDR) allowing only identifications with an FDR of < 5%.

### Gene ontology

Official gene symbols for identified proteins were entered into the gene ontology (GO) databases DAVID (https://david.ncifcrf.gov) and STRING (https://string-db.org). Both these databases were used to identify biological functions and molecular processes which were enriched within our lists of DE proteins.

### MiRNA profiling

Total RNA was extracted from biological duplicate samples collected on day 6 of culture. The NanoDrop ND-1000 spectrophotometer was used to determine RNA quantity and quality. TaqMan Low-Density Array cards (TLDAs) (Human MicroRNA A&B Cards V2.0) were run as per the manufacturer’s guidelines (Applied BioSystems). One hundred nanograms of total RNA was reverse transcribed in two individual multiplex reactions. Twelve cycles of pre-amplification with pre-amp primer pools were then carried out on cDNA mixes. Samples were loaded on to TLDA cards and PCR was performed on a AB7900 real time instrument for 10 min at 95 °C followed by 40 cycles of 30 s at 97 °C and 1 min at 60 °C.

### Evaluating predicted targets of DE miRNAs

Predicted targets of differentially expressed miRNAs were analysed using 3 online miRNA target prediction tools; miRWalk (http://zmf.umm.uni-heidelberg.de/apps/zmf/mirwalk/) TargetScan (http://www.targetscan.org/vert_72/) and miRDb (http://mirdb.org). Both the human and mouse databases were searched using each target prediction database. The lists of predicted targets of differentially expressed miRNAs which were been shown to be DE at the protein level were compiled.

### Cell culture

The CDCL selected for functional analysis were cultured in Lilly propriety maintenance media at 3 × 10^5^ cells/mL and were sub-cultured in E250 mL shake flasks (Helena-BioSciences). Cell were grown in an ISF1-XC Climo-shaker, at 37 °C, 80% humidity and 6% CO_2_. VCD/mL was monitored using the ViaCount^TM^ assay on a Guava® EasyCyte benchtop cytometer (Merck Millipore).

### TransIT-X2 DNA delivery for transfection of miRNA mimics

The TransIT-X2 Dynamic Delivery System (Mirus) was used for transient transfection of miRNA mimics into medium and low Qp CDCLs. Transient transfections were carried out on static cell culture. Cells in mid-exponential phase were seeded in T25 flasks at a concentration of 3 × 10^6^ cells/flask in 5 mL of CHO S SFM II (Gibco) media. Cells were allowed to grow for 24 h. Cells were fed prior to transfection in accordance with the manufacturer’s specifications. IDT miRNA mimic was allowed to complex with CHO S SFM II media and TransIT-X2 (Mirus) reagent for 20–25 min before being added in a dropwise fashion to the appropriate flask. After 20 h cells were passaged, resuspended in 15 mL Lilly propriety media and split into technical triplicates. Cells were grown in 4 mL of Lilly proprietary media in 50 ml spin tubes (Helena-BioSciences) in an ISF1-XC climo-shaker, at 37 °C, 80% humidity and 5% CO_2_. VCD/mL was monitored using the ViaCount™ assay on a Guava® EasyCyte benchtop cytometer for 5 days. On day 5, cell pellets and cell free supernatant samples were taken for further analysis.

### Quantitative reverse-transcription polymerase chain reaction (qRT-PCR)

Cells were harvested on day 5 after transfection by centrifugation at 1000 rpm for 5 min. Tri-reagent (Ambion) was used according to manufacturer’s protocol to isolate total RNA. Total RNA was quantified, and quality was evaluated using NanoDrop (Thermo Fisher Scientific). The TaqMan miRNA Assay® system (Applied Biosystems) was used for miRNA analysis. The Taqman® miRNA Reverse Transcription Kit (Applied Biosystems) was used to perform reverse transcription of specific mature miRNA from total RNA. U6 snRNA was used as an endogenous control. Relative miRNA abundance was determined by qRT-PCR using the ddCt method with U6 snRNA as an endogenous control.

### HPLC quantification of protein

Cell free supernatant media was taken from CHO CDCLs transfected with miRNA mimics or negative control mimics on day 5 of culture and absorbance peaks were obtained. Cell free supernatant was run on an UltiMate 3000 RSLC HPLC using a MAbPacTM RP 4 μm 2.1 × 100 mm column (large pore size polymeric resin) (Thermo Scientific). A flow rate of 500 μL/min, a temperature of 75 °C and an absorbance of 214 nm was applied. The peak obtained represented the amount of full-length product (FLP) present on each day of culture. A standard curve was created using the purified recombinant protein product to determine titer.

### Statistical analysis

A two-tailed student t-test was performed on all functional data and phenotypic assessment of CHO CDCLs. An F-test was performed on all data to determine whether equal or unequal variance should be used for the students t-test. An F statistic of lower value than the critical F value indicated equal variance and an F statistic higher than the critical F value indicated unequal variance. Data with a p-value ≤ 0.05 was considered of low significance, ≤ 0.005 was considered significant and ≤ 0.001 considered of high significance.

## Results and discussion

### MiRNA and LC–MS/MS profiling of high and low Qp CHO CDCLs

The aim of this study was to gain insights into the intracellular mechanisms that contribute to increased/decreased productivity in CHO cells. CHO CDCLs deemed as high Qp were compared to clones deemed as low Qp, and DE miRNAs were identified. Quantitative label free LC–MS/MS proteomic analysis was also carried out on high and low Qp CDCLs and used to identify predicted targets of DE miRNAs which were DE at the protein level. Differential LC–MS/MS profiling allowed us to confidently identify proteins with increased or decreased expression between the two groups. These datasets were used to form a multi-omic assessment of the mechanisms that are contributing to varying levels of Qp in CHO CDCLs. A multi-omic approach to characterise intracellular pathways associated with CHO cell growth rate has previously been carried out (Clarke et al. 2012). This study uses a similar approach while characterising intracellular processes associated with high and low Qp phenotypes. Profiling CHO cell clones at multiple biological levels allows us to form a more comprehensive understanding of CHO cell biology. MiRNAs are known to regulate multiple genes post-transcriptionally; therefore, knowledge of the miRNA, protein and mRNA profiles of CHO CDCLs helps us understand the functional significance of miRNAs and proteins in the cell.

**Fig. 1:**
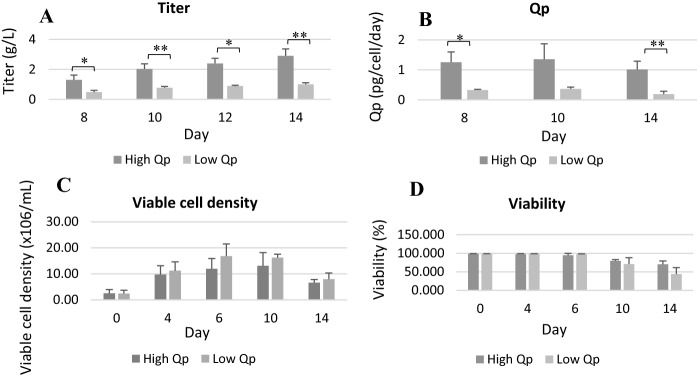
**A** Titer (g/L) of 3 high and 3 low Qp fc-fusion protein producing CHO cell CDCLs on day 8, 10 and 14 of a 14 day shake flask study. Titer was measured via analytical protein A affinity chromatography **B** Cell specific productivity (Qp) high and low Qp CDCLs. **C** Viable cell density (VCD) (× 10^6^cells/mL) of high and low Qp fc-fusion protein producing CHO cell CDCLs on day 0, 4, 6, 10 and 14 of a 14 day shake flask study. **D** Viability of high and low Qp fc-fusion protein producing CHO cell CDCLs on day 0, 4, 6, 10 and 14 of a 14 day shake flask study. Cell density and viability were determined using an automated VicellTM XR cell viability analyser

High and low Fc-fusion protein producing CHO cell CDCLs were grown in a 14-day fed batch shake flask study. High Qp CDCLs were selected based on a high Qp, titer, viability and VCD phenotype, whereas low Qp CDCLs displayed a low Qp and titer with high viability and VCD. CDCLs varied largely in titer at all time-points with titers on day 14 ranging from 2.6 g/L to 2.7 g/L in high Qp CDCLs and from 0.8 g/L to 1.1 g/L in low Qp CDCLs. No statistically significant differences in VCD and viability were observed between high and low Qp CDCLs. MiRNA profiling and LC–MS/MS analysis was carried out on samples taken at day 6, during the exponential growth phase of culture. Day 6 was chosen for profiling due to both high and low Qp CDCLs maintaining a high viability and VCD at this time-point. Viability and VCD begin to decline for all CDCLs at day 10 (Fig. 1).

We identified 15 significantly differentially expressed miRNAs between high and low Qp CDCLs using a fold change cut off 1.2 (Table 1). DE miRNAs were prioritised for further investigation based on their fold change and predicted targets. MiR-200a and miR-878 were found to be highly DE between high and low Qp CDCLs with fold changes of 21.69 and 14.83 respectively. MiR-30e-3p was also prioritised for further investigation, due to a high level of overlap between predicted targets of MiR-30e-3p and targets identified with decreased protein expression in high Qp CDCLs. Quantitative label free LC–MS/MS analysis was used to gain an overall view of DE proteins between high and low Qp CHO CDCLs. GO databases DAVID (https://david.ncifcrf.gov) and STRING (https://string-db.org) were used to identify biological processes and molecular functions which were associated with the DE proteins between high and low Qp CDCLs. Table 2 shows a list of highly enriched biological processes between high and low Qp CDCLs at day 6. In total, 356 proteins were identified as DE between high and low Qp CDCLs, with 138 protein having increased expression in high Qp CDCLs and 217 proteins with increased expression in low Qp CDCLs (Supplementary Table 1). Protein folding was the most significantly enriched biological process shared between high and low Qp CDCLs. The majority of protein folding associated proteins identified (21 out of 26) were found to have decreased expression in high Qp CDCLs (Supplementary Table 2). An enrichment of proteins associated with response to stress, UPR and chaperone mediated protein folding was found in the list of proteins with decreased expression in high Qp CDCLs. Future work could involve the use of targeted mass spectrometry based approaches such as Parallel Reaction Monitoring for quantification of protein expression changes of target proteins (Abelin et al. 2016).

**Table 1:** Differentially expressed miRNAs identified during miRNA profiling (with fold changes and P values)

miRNA	Phenotype	Fold change	P value
miR-378	Up in high Qp	2.42	0.004
miR-30e-3p	Up in high Qp	1.59	0.009
miR-138	Up in high Qp	2.03	0.013
miR-374-5p	Up in high Qp	2.36	0.017
miR-202-3p	Up in high Qp	2.33	0.022
miR-184	Up in high Qp	3.61	0.023
let-7f	Up in high Qp	1.43	0.027
miR-24–2#	Up in high Qp	1.54	0.033
miR-384-3p	Up in low Qp	10.04	0.036
miR-878	Up in high Qp	14.83	0.037
snoRNA-135	Up in high Qp	1.37	0.038
miR-200a	Up in high Qp	21.69	0.040
miR-140	Up in high Qp	1.43	0.040
miR-190b	Up in high Qp	1.98	0.042
miR-22#	Up in high Qp	2.51	0.045

**Table 2:** Biological processes enriched in high Vs low Qp CDCLs at day 6 (https://david.ncifcrf.gov)

David category	Biological Process	P-value	Benjamini
GOTERM_BP_DIRECT	Protein folding	1.10E-14	1.80E-11
GOTERM_BP_DIRECT	Cell–cell adhesion	1.60E-08	1.40E-05
GOTERM_BP_DIRECT	Carbohydrate metabolic process	4.90E-08	2.80E-05
GOTERM_BP_DIRECT	Response to endoplasmic reticulum stress	1.70E-06	7.50E-04
GOTERM_BP_DIRECT	Chaperone-mediated protein folding	6.00E-06	2.10E-03
GOTERM_BP_DIRECT	Cholesterol biosynthetic process	7.20E-06	2.10E-03
GOTERM_BP_DIRECT	IRE1-mediated unfolded protein response	1.70E-05	4.20E-03
GOTERM_BP_DIRECT	mRNA splicing, via spliceosome	1.10E-04	2.40E-02
GOTERM_BP_DIRECT	Mitochondrion organization	1.20E-04	2.30E-02
GOTERM_BP_DIRECT	Gluconeogenesis	1.90E-04	3.20E-02
GOTERM_BP_DIRECT	Tricarboxylic acid cycle	2.10E-04	3.30E-02
GOTERM_BP_DIRECT	Glycosphingolipid metabolic process	2.20E-04	3.10E-02
GOTERM_BP_DIRECT	Cell redox homeostasis	7.20E-04	9.10E-02
GOTERM_BP_DIRECT	Protein deglycosylation	7.80E-04	9.20E-02
GOTERM_BP_DIRECT	Response to drug	9.00E-04	9.80E-02
GOTERM_BP_DIRECT	Response to ethanol	9.90E-04	1.00E-01
GOTERM_BP_DIRECT	Protein N-linked glycosylation via asparagine	9.90E-04	9.60E-02
GOTERM_BP_DIRECT	ER-associated ubiquitin-dependent protein catabolic process	1.00E-03	9.50E-02

### Evaluating predicted targets of DE miRNAs

MiR-878 and miR-30e-3p were found to have increased expression in high Qp CDCLs by 14.83 fold and 1.59 fold respectively. We identified 11 predicted targets of miR-878 which were shown have decreased protein expression in the high Qp CDCLs. Data relating to the mRNA profile of this panel of Fc-fusion protein producing CHO CDCLs, which was reported in a previous study (Clarke et al. 2019) was also taken into consideration. Of the 11 predicted targets identified, 8 were also found to have no detected change at the mRNA level, making them strong candidates as targets of miR-878. GO analysis identified an enrichment of proteins associated with protein folding related pathways such as the ubiquitin dependent ERAD pathway (ERLIN2, SEL1L, STT3B) (Supplementary Table 4). We identified 87 predicted targets of miR-30e-3p with decreased protein expression in high Qp CDCLs, 81 were also found to have no detected change at the mRNA level. GO analysis identified an enrichment of proteins associated with protein folding, protein secretion and the IRE1 mediated UPR (Supplementary Table 5). MiR-200a expression was also found to be increased in high Qp CDCLs, with a fold change of 21.69, the highest fold change observed in the DE miRNAs. We identified 56 predicted targets of miR-200a which were shown to have decreased protein expression in high Qp CDCLs. Of the 56 predicted targets identified, 48 were also found to have no detected change at the mRNA level. We found UPR to be an enriched biological process within this list and included proteins such as ATP6V0D1, HSP90B1, HYOU1, PDIA5, TRAP1 and TPP1 (Supplementary Table 3).

**Fig. 2:**
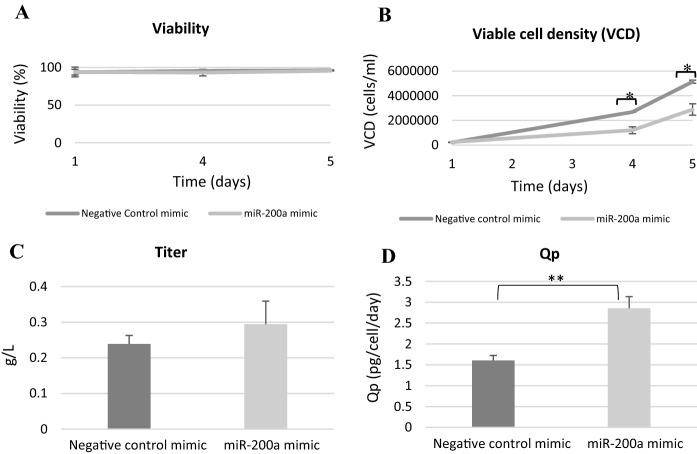
**A** Viability **B** Viable cell density **C** Titer and **D** Qp of a medium Qp CDCLs transfected with a negative control mimic and a miR-200a mimic, values based on the mean of three biological replicates with error as standard deviation of replicates

**Fig. 3:**
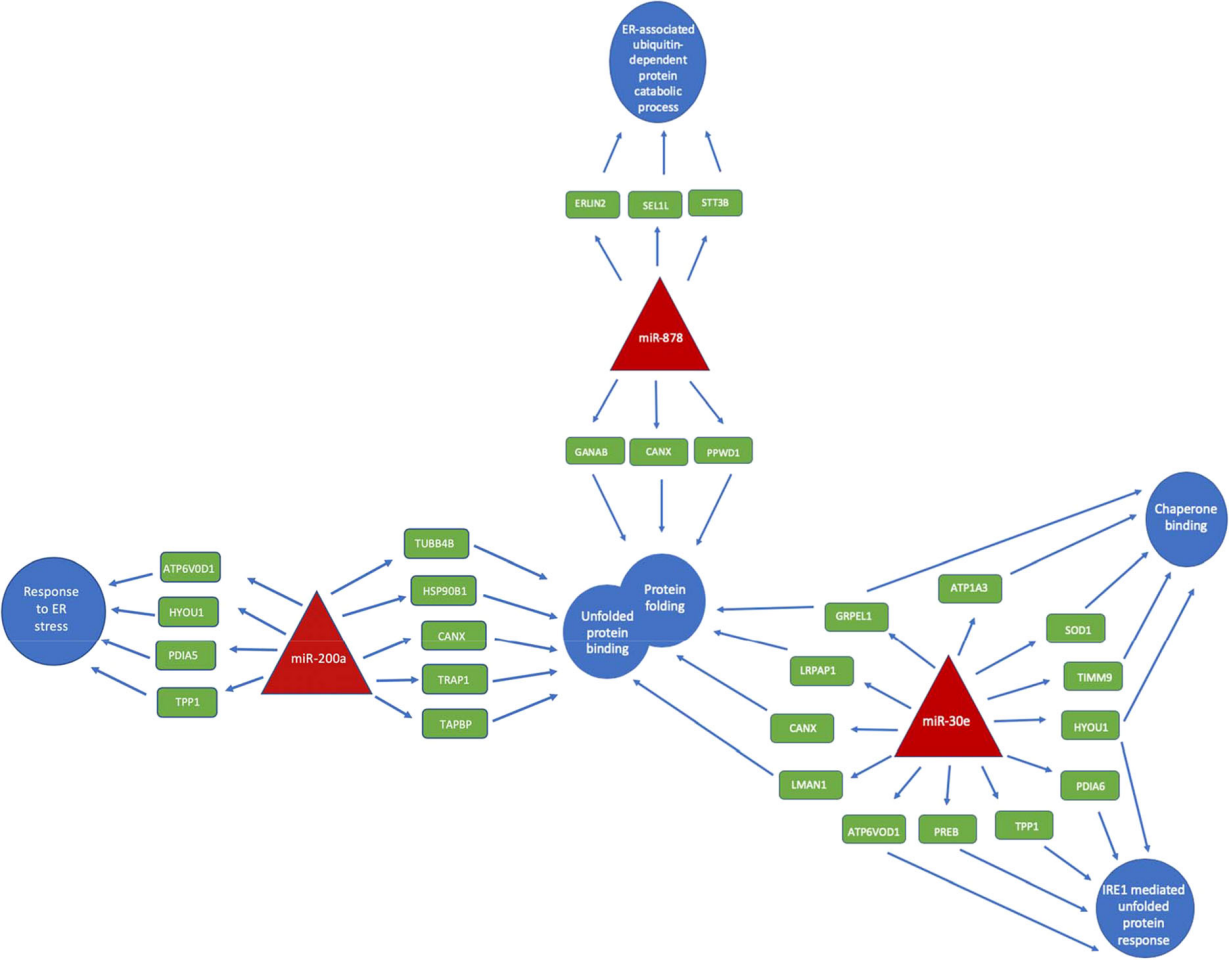
Red triangles represent DE miRNAs. Green rectangles show predicted targets of miRNAs which are down in high Qp CDCLs at protein level and have no detected change mRNA level. Blue circles represent biological processes associated with these proteins

**Fig. 4:**
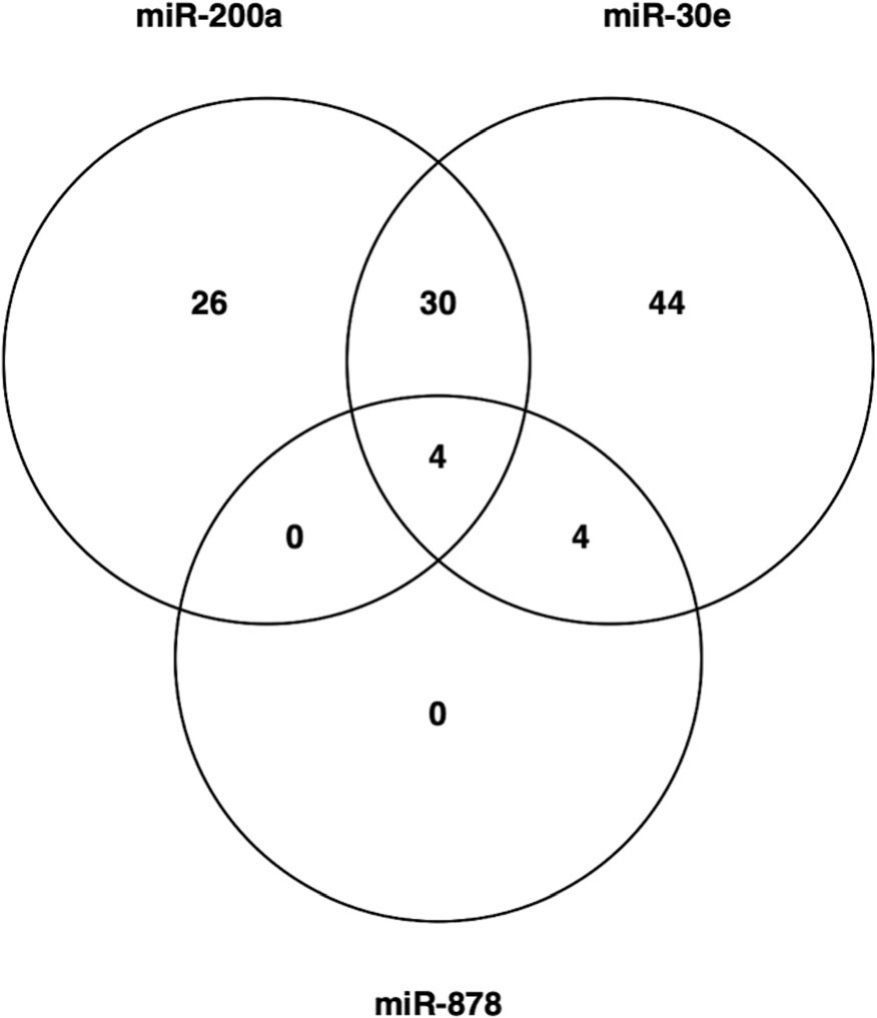
Overlap in predicted targets of miR-200a, miR-30e and miR-878. These targets were also DE between high and low Qp CDCLs at the protein level. The full table of overlapping targets is shown in Supplementary Table 6

MiR-200a, miR-30e-3p and miR-878 were chosen for functional investigation due to the fold change between high and low Qp CDCLs and their predicted targets. MiRNA mimics were transfected into a CHO cell line chosen from the panel of CDCLs. The cell line chosen for functional analysis displayed a medium Qp and titer (0.68 pg/cell/day and 0.1 g/L at day 8 of culture) and a high viability and VCD. This phenotype was chosen to allow functional work to focus on improving titer and Qp. The viability and VCC of the cells was monitored after transfection of miRNA mimics. Cell free supernatant was collected at day 5 after transfection and was analysed by HPLC. None of the targets chosen for functional validation demonstrated a significant effect on viability (Fig. 2A and Supplementary Fig. 1A). Overexpression of miR-30e-3p and miR-878 using miRNA mimics yielded no significant increase or decrease in titer or Qp (Supplementary Fig. 1). Overexpression of miR-200a using a miRNA mimic resulted in a reduction in VCD (Fig. 2B). MiR-200a overexpression also caused a 1.2 fold increase in titer (Fig. 2C) and a significant 1.8 fold increase in Qp (Fig. 2D). Although miR-878 and miR-30e-3p did not have a functional effect on titer or Qp when investigated, the differential expression of these miRNAs and their predicted protein targets still presents valuable information. As we know miRNAs are dynamic and often target multiple genes allowing for large-scale post-transcriptional regulation of genes. These results could show how multiple miRNAs work together to target a common pathway in the cell. All three DE miRNAs were found to have several predicted targets associated with protein folding and/or UPR, which have been shown to have decreased expression in high Qp CDCLs at the protein level and have no detected change at the mRNA level such as CANX, GANAB and HSP90AB1 (Fig. 3). Several protein were also found to be common targets of miR-200a, miR-30e and miR-878 such as CANX which is a target of all 3 miRNA or HYOU1 which is a target of miR-200a and miR-30e (Fig. 4). The majority of the protein folding associated proteins identified with decreased expression in high Qp CDCLs were associated with protein folding as a response to stress. Levels of cellular stress may be lower in high Qp CDCLs, resulting in decreased expression of these proteins and more efficient protein translation.

### MiR-200a

In this study miR-200a was identified as having significantly increased expression in high Qp CDCLs. Functional investigation showed that overexpression of miR-200a in a CHO cell line resulted in a reduction of VCD and an increase in titer and Qp. These results indicate miR-200a may be a good target for cell engineering to increase titer/Qp. Previous studies have shown that miR-200a is often downregulated in cancer (Xia et al. 2010; van Kempen et al. 2012; Hill et al. 2013). For example, miR-200a was found to be downregulated in relapsed prostate cancer patients after prostectomy (Barron et al. 2012).

We identified that a number of proteins associated with the UPR which are predicted targets of miR-200a, have decreased expression in the high Qp CDCLs at the protein level, and had no detected change at the mRNA level (ATP6V0D1, HSP90B1, HYOU1, PDIA5, TPP1, TRAP1). These results may suggest a role for miR-200a in post-transcriptionally regulating UPR expression in CHO cells. The UPR is one of three pathways responsible for maintaining homeostasis in the ER, along with the endoplasmic reticulum associated folding (ERAF) pathway and the ER associated protein degradation ERAD pathway (Schroder 2008). These signalling pathways are activated as a response to ER stress. The UPR pathway acts by stopping protein translation, thereby reducing the burden on the cell, to help maintain homeostasis. The level of folding possible in the cell can also be enhanced by increasing the levels of transcription of certain chaperones (Schroder and Kaufman 2005; Schroder 2006). Endoplasmin (HSP90B1), also known as GRP94, is a UPR associated protein which was found to have decreased expression in high Qp CDCLs and is a predicted target of miR-200a. HSP90B1 is a ER chaperone and has been shown to be associated with protein folding particularly in response to ER stress. Hypoxia upregulated 1 (HYOU1) also known as 150 kDa oxygen-regulated protein (ORP150) or GRP170 is also a UPR associated protein and a predicted target of miR-200a which was shown to have decreased expression in high Qp CDCLs at the protein level. HYOU1 also have no detected change at the mRNA level. HYOU1 is a member of the HSP70 family of proteins and is important for cytoprotection during hypoxia induced cellular perturbation. Studies have shown that HYOU1 binds directly to unfolded Ig subunits and also binds to secreted substrates although to a lesser extent, suggesting it plays a role as a chaperone in the cell (Behnke and Hendershot 2014). Protein disulfide-isomerase A5 (PDIA5) was also identified as a UPR associated protein. PDIA5 was shown to be 4.19 fold downregulated in the high Qp CDCLs, have no detected change at the mRNA level and is also a predicted target of miR-200a. PDIA5 is a member of the protein disulfide isomerase (PDI) family. PDIs function as folding catalysts by accelerating the chemical steps which accompany protein folding (Gilbert 1997). Protein folding is highly prone to errors. Disulfides can often be formed in the wrong order and therefore must be rearranged in the correct order to allow protein folding to continue. PDIs are responsible for correcting these mistakes by replacing incorrect disulfides with the correct ones (Todd et al. 1996; Gilbert 1997).

**Fig. 5:**
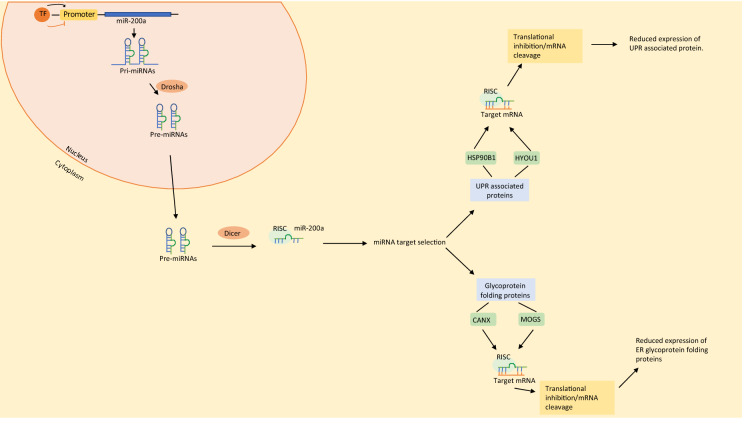
Schematic illustration of potential miR-200a mediated regulation of UPR and glycoprotein folding associated protein. The miRNA is formed in the nucleus of the cell. Mir-200a genes are transcribed by Pol II to produce pri-miRNAs. Dicer cleaves pre-miRNA in the cytoplasm producing a 22 base pair miRNA duplex. The miRNA RISC is formed and recognises the mRNA target. Binding of mature miR-200a to the mRNA target (UPR associated protein/glycoprotein folding protein) results in translational repression or mRNA cleavage

Attempts to target UPR associated proteins in CHO cells have produced varied results depending on cell line, protein product and target genes. Overexpression of PDI in CHO cells led to an increase in the secretion of human antibodies (Borth et al. 2005). However, this was not the case for TNFR:Fc which experienced intracellular retention upon overexpression of PDI (Davis et al. 2000). The results presented in this study could suggest that increased miR-200a regulated post-transcriptional regulation of the UPR results in increased recombinant protein production. Figure 5 illustrate how miR-200a could potentially post-transcriptionally regulate the UPR along with other protein folding associated proteins. Although previous attempts to target the UPR in CHO cells have produced varied responses, perhaps altering miR-200a expression in order to post-transcriptionally target the UPR is a more effective method of targeting the UPR to increase recombinant protein production. Altering miR-200a expression may allow us to alter the expression of multiple UPR associated genes at once which could create optimal intracellular conditions for high recombinant protein production. Future work investigating miR-200a overexpression in CHO cells will involve carrying out miR-200a overexpression across a panel of CHO cell lines to determine whether effects on titer and Qp are cell line specific. Additional studies would also involve differential LC–MS/MS analysis of CHO CDCLs in which miR-200a has been overexpressed in order to identify changes in expression of UPR associated proteins.

We also identified a number of proteins associated with glycoprotein folding with decreased expression in high Qp CDCLs, which have no detected change at the mRNA level. CANX protein expression was found to be 1.84 fold decreased in high Qp CDCLs and have no detected change at the mRNA level. CANX is a predicted target of miR-200a and plays an important role in the maturation of Glycosylphosphatidylinositol (GPI) anchors, including the binding of N-glycans on GPI anchors which induces protein folding. GPI anchors are understood to modify several cell surface proteins in the ER (Fujita and Kinoshita 2012). CANX is also involved in keeping GPI anchors in the ER and helps maintain their efficient inositol-deacylation by post-GPI attachment to proteins 1 (PGAP1) (Liu et al. 2018). Mannosyl-oligosaccharide glucosidase (MOGS) and neutral alpha-glucosidase AB (GANAB) were also found to have decreased protein expression in high Qp CDCLs. MOGS is a predicted target of miR-200a. Although GANAB is not a predicted target of miR-200a it was found to be a predicted target of miR-30e-3p and miR-878. MOGS, GANAB and CANX all play a role in the same calnexin cycle in order to monitor glycoprotein folding (Helenius and Aebi 2004; Tannous et al. 2015; Lamriben et al. 2016). Studies have shown that when these three genes are disturbed, newly synthesised proteins are inhibited from entering the calnexin cycle (Liu et al. 2018). These results suggest a reduction in glycoprotein folding in high Qp CDCLs. This could be due to the cell focussing its folding machinery on correct folding of the recombinant protein product in high Qp CDCLs.

## Conclusion

The aim of this study was to characterise intracellular pathways affecting Qp using miRNA and proteomic profiling of high and low Qp CHO cell CDCLs. Results showed that several miRNAs upregulated in high Qp CDCLs are predicted to target proteins associated with protein folding and that the majority of these proteins have decreased expression in high Qp CDCLs and have no detected change at the mRNA level. Overall this data suggests that these miRNAs may play a role in supressing expression of proteins associated with corrective protein folding in the high Qp CDCLs and the downregulation of these miRNAs in low Qp CDCLs may allow for high expression of protein folding associated proteins in an attempt to correct improper protein folding in the low Qp CDCLs. Mir-200a was found to target a number of proteins associated with the UPR. Functional investigation showed overexpression of miR-200a resulted in decreased VCD, a 1.2 fold increase in titer and a 1.8 fold increase in Qp. These results may suggest a role for miR-200a in the post-transcriptional regulation of the UPR and presents miR-200a as a potential target for cellular engineering of CHO cells in order to optimize protein production. MiR-200a expression was shown to be correlated with both cell growth and Qp. Although high cell growth rates are extremely desirable during the exponential phase of growth, a reduced cell growth rate in combination with high levels of protein production is highly desirable during the stationary phase of growth. In an inducible expression system, miR-200a overexpression could be used to reduce cell growth towards the stationary phase while enhancing protein output. This would result in a lower cell volume for downstream purification, greater protein output and possibly less cell death due to a reduced VCD.

## Supplementary Information

Below is the link to the electronic supplementary material.

Supplementary file1 (XLSX 36 kb)

Supplementary file2 (DOCX 98 kb)

## Declarations

### Research involving human and animal participants

This article does not contain any studies with human participants or animals performed by any of the authors.
