# Supplementary material for: Differential expression of miRNAs and functional role of mir-200a in high and low productivity CHO cells expressing an Fc fusion protein
Source: Biotechnol Lett. 2021 Jun 16;43(8):1551–63. doi: 10.1007/s10529-021-03153-7 (PMC8254715; doi:10.1007/s10529-021-03153-7)
Supplement: Supplementary file 2 — Supplementary file2 (DOCX 98 kb) [file 10529_2021_3153_MOESM2_ESM.docx]

**Differential expression of miRNAs and functional role of mir-200a in high and low productivity CHO cells expressing an Fc fusion protein.**

Laura Bryan^a^, Michael Henry^a^, Niall Barron^a^, Clair Gallagher^a^, Ronan M. Kelly^b^, Christopher C. Frye^b^, Matthew D. Osborne^c^, Martin Clynes^a,^, Paula Meleady^a,^.

^a^ National Institute for Cellular Biotechnology, Dublin City University, Glasnevin, Dublin 9, Ireland

^b^ Eli Lilly and Company, LTC-North, 1200 Kentucky Avenue, Indianapolis, IN, 46225, United States

^c^ Eli Lilly S.A. Irish Branch, Kinsale, Cork, Ireland

Corresponding author: Laura Bryan ([laura.bryan2@mail.dcu.ie](mailto:laura.bryan2@mail.dcu.ie))

Biotechnology Letters Section: Bioprocessing and Bioengineering

### Supplementary data.

| Accession | Gene name | Description | Anova (p) | Max fold change | Protein phenotype | Biological Process |
| --- | --- | --- | --- | --- | --- | --- |
| G3I3Z1 | AIP | AH receptor-interacting protein | 0.02185475 | 2.23 | Up in high Qp | Protein folding |
| G3I8V9 | DNAJA1 | DnaJ-like subfamily A member 1 | 0.02523686 | 1.76 | Up in high Qp | Protein folding |
| G3ICC2 | BAG3 | BAG family molecular chaperone regulator 3 | 0.02830967 | 1.52 | Up in high Qp | Protein folding |
| G3HIJ0 | GNAI3 | Guanine nucleotide-binding protein G(O) subunit alpha (Fragment) | 0.02737644 | 2.01 | Up in high Qp | Protein folding |
| G3HYP6 | NUDC | Nuclear migration protein nudC | 0.00192313 | 1.69 | Up in high Qp | Protein folding |
| G3HEY8 | SIL1 | Nucleotide exchange factor SIL1 | 0.00404298 | 5.42 | Down in high Qp | Protein folding |
| G3GSP9 | PDIA5 | Protein disulfide-isomerase A5 | 0.00064001 | 4.19 | Down in high Qp | Protein folding |
| G3HMQ0 | PPWD1 | Peptidylprolyl isomerase | 0.01297639 | 2.39 | Down in high Qp | Protein folding |
| G3HB04 | PDIA6 | Protein disulfide-isomerase A6 | 0.00432231 | 2.29 | Down in high Qp | Protein folding |
| G3HQM6 | HSP90B1 | Endoplasmin | 0.00501733 | 2.26 | Down in high Qp | Protein folding |
| G3I027 | TRAP1 | Heat shock protein 75 kDa, mitochondrial | 0.02737504 | 1.98 | Down in high Qp | Protein folding |
| G3HCX8 | CALR3 | Calreticulin | 0.03046012 | 1.87 | Down in high Qp | Protein folding |
| G3HP69 | CANX | Calnexin | 0.01866521 | 1.84 | Down in high Qp | Protein folding |
| G3HRG8 | ERP44 | Endoplasmic reticulum resident protein ERp44 (Fragment) | 0.03472767 | 1.80 | Down in high Qp | Protein folding |
| G3HLV1 | FKBP10 | Peptidylprolyl isomerase | 0.01520222 | 1.80 | Down in high Qp | Protein folding |
| G3GWB3 | LRPAP1 | Alpha-2-macroglobulin receptor-associated protein | 0.02213005 | 1.77 | Down in high Qp | Protein folding |
| G3GWC4 | GRPEL1 | GrpE protein homolog | 0.00495017 | 1.75 | Down in high Qp | Protein folding |
| G3HUI7 | LMAN1 | Protein ERGIC-53 | 0.00339396 | 1.65 | Down in high Qp | Protein folding |
| G3HYG7 | DNAJB11 | DnaJ-like subfamily B member 11 | 0.04033002 | 1.63 | Down in high Qp | Protein folding |
| G3I064 | GANAB | Neutral alpha-glucosidase AB | 0.00489893 | 1.87 | Down in high Qp | Protein folding |
| G3HI57 | MLEC | Malectin | 0.00324968 | 1.63 | Down in high Qp | Protein folding |
| G3I7V6 | MOGS | Mannosyl-oligosaccharide glucosidase | 0.04299644 | 3.69 | Down in high Qp | Protein folding |
| G3I9P1 | MESDC2 | LDLR chaperone MESD | 0.02579659 | 1.9 | Down in high Qp | Protein folding |
| G3H0U6 | PDIA3 | Protein disulfide-isomerase A3 | 0.01860903 | 3.44 | Down in high Qp | Protein folding |
| G3IDT6 | PDIA4 | Protein disulfide-isomerase A4 | 0.02257604 | 2.38 | Down in high Qp | Protein folding |
| G3H697 | PRKCSH | Glucosidase 2 subunit beta | 0.00073965 | 1.59 | Down in high Qp | Protein folding |

Supplementary Table 2: Proteins associated with protein folding which were found to be differentially expressed between high and low Qp at day 6.

| MiRNA | MiRNA phenotype | Predicted gene target | Description | Protein phenotype | Fold Change | mRNA phenotype | Biological process |
| --- | --- | --- | --- | --- | --- | --- | --- |
| miR-200a-3p | Up in high Qp | ABCC8 | ATP-binding cassette sub-family D member 3 (Fragment) | Down in high Qp | 2.09 | Unchanged |  |
| miR-200a-3p | Up in high Qp | ACAD11 | Acyl-CoA dehydrogenase family member 11 | Down in high Qp | 1.92 | Unchanged |  |
| miR-200a-3p | Up in high Qp | ACTA2 | Actin, aortic smooth muscle | Down in high Qp | 2.05 | Unchanged |  |
| miR-200a-3p | Up in high Qp | ADPGK | ADP-dependent glucokinase | Down in high Qp | 3.08 | Down in high Qp |  |
| miR-200a-3p | Up in high Qp | ANXA5 | Annexin | Down in high Qp | 1.79 | Unchanged |  |
| miR-200a-3p | Up in high Qp | APLP2 | Amyloid-like protein 2 | Down in high Qp | 3.84 | Unchanged |  |
| miR-200a-3p | Up in high Qp | ASPH* | Aspartyl/asparaginyl beta-hydroxylase (Fragment) | Down in high Qp | 2.11 | Unchanged |  |
| miR-200a-3p | Up in high Qp | ATP6V0D1 | V-type proton ATPase subunit | Down in high Qp | 1.61 | Unchanged | UPR |
| miR-200a-3p | Up in high Qp | ATP6V1A* | V-type proton ATPase catalytic subunit A | Down in high Qp | 1.51 | Unchanged |  |
| miR-200a-3p | Up in high Qp | CAMK2G | Calcium/calmodulin-dependent protein kinase type II gamma chain | Down in high Qp | 1.52 | Unchanged |  |
| miR-200a-3p | Up in high Qp | CANX* | Calnexin | Down in high Qp | 1.84 | Unchanged | Protein folding |
| miR-200a-3p | Up in high Qp | CHID1 | Chitinase domain-containing protein 1 | Down in high Qp | 2.33 | Unchanged |  |
| miR-200a-3p | Up in high Qp | COLGALT1 | Glycosyltransferase 25 family member 1 | Down in high Qp | 2.21 | Down in high Qp |  |
| miR-200a-3p | Up in high Qp | CTSB | Cathepsin B | Down in high Qp | 1.83 | Unchanged |  |
| miR-200a-3p | Up in high Qp | CYR61 | Protein CYR61 | Down in high Qp | 19.53 | Unchanged |  |
| miR-200a-3p | Up in high Qp | DDOST* | Dolichyl-diphosphooligosaccharide--protein glycosyltransferase 48 kDa subunit | Down in high Qp | 1.89 | Unchanged |  |
| miR-200a-3p | Up in high Qp | DYNLL2 | Dynein light chain | Down in high Qp | 1.69 | Unchanged |  |
| miR-200a-3p | Up in high Qp | ERMP1 | Endoplasmic reticulum metallopeptidase 1 | Down in high Qp | 3.34 | Unchanged |  |
| miR-200a-3p | Up in high Qp | FAM114A1 | Protein Noxp20 (Fragment) | Down in high Qp | 1.85 | Unchanged |  |
| miR-200a-3p | Up in high Qp | FBXL20* | F-box/LRR-repeat protein 2 | Down in high Qp | 2.26 | Unchanged |  |
| miR-200a-3p | Up in high Qp | FKBP10 | Peptidylprolyl isomerase | Down in high Qp | 1.80 | Down in high Qp | Protein folding |
| miR-200a-3p | Up in high Qp | FLOT1 | Flotillin-1 | Down in high Qp | 2.63 | Unchanged | ER stress response |
| miR-200a-3p | Up in high Qp | FUCA1 | Tissue alpha-L-fucosidase | Down in high Qp | 2.55 | Unchanged |  |
| miR-200a-3p | Up in high Qp | GALNT2 | Polypeptide N-acetylgalactosaminyltransferase | Down in high Qp | 1.67 | Unchanged |  |
| miR-200a-3p | Up in high Qp | GLA | Alpha-galactosidase A | Down in high Qp | 4.11 | Unchanged |  |
| miR-200a-3p | Up in high Qp | GPD1 | Glycerol-3-phosphate dehydrogenase [NAD(+)] | Down in high Qp | 1.56 | Unchanged |  |
| miR-200a-3p | Up in high Qp | GSN | Gelsolin | Down in high Qp | 1.84 | Unchanged |  |
| miR-200a-3p | Up in high Qp | HPSE | Heparanase | Down in high Qp | 8.57 | Unchanged |  |
| miR-200a-3p | Up in high Qp | HSP90B1 | Endoplasmin | Down in high Qp | 2.26 | Unchanged | Protein folding/ UPR/ ER stress response |
| miR-200a-3p | Up in high Qp | HYOU1 | Hypoxia up-regulated protein 1 | Down in high Qp | 2.07 | Unchanged | UPR/ ER stress response |
| miR-200a-3p | Up in high Qp | IGF2R | Cation-independent mannose-6-phosphate receptor | Down in high Qp | 2.55 | Down in high Qp |  |
| miR-200a-3p | Up in high Qp | ITGAV | Integrin alpha-V | Down in high Qp | 2.58 | Unchanged |  |
| miR-200a-3p | Up in high Qp | KCNAB2 | Voltage-gated potassium channel subunit beta-2 | Down in high Qp | 1.82 | Unchanged |  |
| miR-200a-3p | Up in high Qp | LAMA5 | Laminin subunit alpha-5 | Down in high Qp | 9.85 | Down in high Qp |  |
| miR-200a-3p | Up in high Qp | LAMB1 | Laminin subunit beta-1 | Down in high Qp | 1.92 | Unchanged |  |
| miR-200a-3p | Up in high Qp | LBR* | Lamin-B receptor | Down in high Qp | 1.74 | Unchanged |  |
| miR-200a-3p | Up in high Qp | MAN2A1 | Alpha-mannosidase 2 | Down in high Qp | 1.61 | Unchanged |  |
| miR-200a-3p | Up in high Qp | MDH2 | Malate dehydrogenase (Fragment) | Down in high Qp | 1.58 | Unchanged |  |
| miR-200a-3p | Up in high Qp | MOGS | Mannosyl-oligosaccharide glucosidase | Down in high Qp | 3.69 | Down in high Qp | Protein folding |
| miR-200a-3p | Up in high Qp | MTA2 | Metastasis-associated protein MTA2 | Down in high Qp | 1.71 | Unchanged |  |
| miR-200a-3p | Up in high Qp | PCOLCE | Procollagen C-endopeptidase enhancer 1 | Down in high Qp | 7.97 | Unchanged |  |
| miR-200a-3p | Up in high Qp | PDIA5 | Protein disulfide-isomerase A5 | Down in high Qp | 4.19 | Unchanged | Protein folding/ UPR/ ER stress response |
| miR-200a-3p | Up in high Qp | PEX14 | Peroxisomal membrane protein PEX14 (Fragment) | Down in high Qp | 1.70 | Unchanged |  |
| miR-200a-3p | Up in high Qp | PLD3 | Phospholipase D3 | Down in high Qp | 1.92 | Unchanged |  |
| miR-200a-3p | Up in high Qp | PPWD1 | Peptidylprolyl isomerase | Down in high Qp | 2.39 | Unchanged | Protein folding |
| miR-200a-3p | Up in high Qp | PXDN | Peroxidasin-like | Down in high Qp | 3.81 | Unchanged |  |
| miR-200a-3p | Up in high Qp | RPN1 | Dolichyl-diphosphooligosaccharide--protein glycosyltransferase subunit 1 | Down in high Qp | 1.80 | Unchanged |  |
| miR-200a-3p | Up in high Qp | SEC23IP | SEC23-interacting protein | Down in high Qp | 1.71 | Unchanged |  |
| miR-200a-3p | Up in high Qp | SEMA3E | Semaphorin-3E | Down in high Qp | 77.41 | Unchanged |  |
| miR-200a-3p | Up in high Qp | SERPINH1* | Serpin H1 | Down in high Qp | 3.22 | Down in high Qp |  |
| miR-200a-3p | Up in high Qp | SHMT2* | Serine hydroxymethyltransferase | Down in high Qp | 1.51 | Down in high Qp |  |
| miR-200a-3p | Up in high Qp | SMC3 | Structural maintenance of chromosomes protein 3 | Down in high Qp | 1.51 | Unchanged |  |
| miR-200a-3p | Up in high Qp | SRP72 | Signal recognition particle 72 kDa protein | Down in high Qp | 1.80 | Unchanged |  |
| miR-200a-3p | Up in high Qp | STAT3 | Signal transducer and activator of transcription | Down in high Qp | 1.65 | Unchanged |  |
| miR-200a-3p | Up in high Qp | SYNJ2BP | Synaptojanin-2-binding protein | Down in high Qp | 1.81 | Unchanged |  |
| miR-200a-3p | Up in high Qp | TAP2 | Antigen peptide transporter 2 | Down in high Qp | 1.91 | Unchanged |  |
| miR-200a-3p | Up in high Qp | TAPBP | Tapasin | Down in high Qp | 1.89 | Unchanged |  |
| miR-200a-3p | Up in high Qp | TGM2 | Protein-glutamine gamma-glutamyltransferase 2 | Down in high Qp | 3.05 | Unchanged |  |
| miR-200a-3p | Up in high Qp | TIMM44 | Mitochondrial import inner membrane translocase subunit TIM44 | Down in high Qp | 1.52 | Unchanged |  |
| miR-200a-3p | Up in high Qp | TMTC3 | Transmembrane and TPR repeat-containing protein 3 | Down in high Qp | 3.35 | Unchanged |  |
| miR-200a-3p | Up in high Qp | TPP1 | Tripeptidyl-peptidase 1 | Down in high Qp | 2.17 | Unchanged | UPR |
| miR-200a-3p | Up in high Qp | TRAP1 | Heat shock protein 75 kDa, mitochondrial | Down in high Qp | 1.98 | Unchanged | UPR |
| miR-200a-3p | Up in high Qp | TUBB4B | Tubulin beta chain | Down in high Qp | 1.84 | Unchanged |  |

Supplementary Table 3: Predicted targets of miR-200a-3p which were shown to be down in high Qp at protein level at day 6. (* indicates the protein was identified as a predicted target of the miRNA by >3 databases).

| MiRNA | MiRNA phenotype | Predicted gene target | Description | Protein phenotype | Fold Change | mRNA phenotype | Biological Process |
| --- | --- | --- | --- | --- | --- | --- | --- |
| miR-878-5p | Up in high Qp | Acbd5 | Acyl-CoA-binding domain-containing protein 5 | Down in high Qp | 1.80 | Unchanged |  |
| miR-878-5p | Up in high Qp | Acad11 | Acyl-CoA dehydrogenase family member 11 | Down in high Qp | 1.92 | Unchanged |  |
| miR-878-5p | Up in high Qp | Canx | Calnexin | Down in high Qp | 1.84 | Unchanged |  |
| miR-878-5p | Up in high Qp | Erlin2 | Erlin-2 | Down in high Qp | 2.11 | Unchanged | ERAD Pathway |
| miR-878-5p | Up in high Qp | Ganab | Neutral alpha-glucosidase AB | Down in high Qp | 1.87 | Down in high Qp |  |
| miR-878-5p | Up in high Qp | Ppwd1 | Peptidylprolyl isomerase | Down in high Qp | 2.39 | Unchanged |  |
| miR-878-5p | Up in high Qp | Uggt1 | UDP-glucose:glycoprotein glucosyltransferase 1 | Down in high Qp | 2.30 | Down in high Qp |  |
| miR-878-5p | Up in high Qp | Sel1l | Protein sel-1-like 1 | Down in high Qp | 3.31 | Unchanged | ERAD Pathway |
| miR-878-5p | Up in high Qp | Sema3e | Semaphorin-3E | Down in high Qp | 77.41 | Unchanged |  |
| miR-878-5p | Up in high Qp | Serpinh1 | Serpin H1 | Down in high Qp | 3.22 | Down in high Qp |  |
| miR-878-5p | Up in high Qp | Stt3b | Dolichyl-diphosphooligosaccharide--protein glycosyltransferase subunit STT3B | Down in high Qp | 1.76 | Unchanged | ERAD Pathway |

Supplementary Table 4: Predicted targets of miR-878-5p which were shown to be DE at protein level at day 6. All proteins were identified as predicted targets.

| MiRNA | MiRNA phenotype | Predicted gene target | Description | Protein phenotype | Fold Change | mRNA phenotype |  |
| --- | --- | --- | --- | --- | --- | --- | --- |
| miR-30e-3p | Up in high Qp | ABCC8 | ATP-binding cassette sub-family D member 3 (Fragment) | Down in high Qp | 2.09 | Unchanged |  |
| miR-30e-3p | Up in high Qp | ACAD11* | Acyl-CoA dehydrogenase family member 11 | Down in high Qp | 1.92 | Unchanged |  |
| miR-30e-3p | Up in high Qp | ACBD5* | Acyl-CoA-binding domain-containing protein 5 | Down in high Qp | 1.80 | Unchanged |  |
| miR-30e-3p | Up in high Qp | ACTA2 | Actin, aortic smooth muscle | Down in high Qp | 2.05 | Unchanged |  |
| miR-30e-3p | Up in high Qp | ADAM10* | Disintegrin and metalloproteinase domain-containing protein 10 | Down in high Qp | 2.37 | Unchanged | Secretion |
| miR-30e-3p | Up in high Qp | APLP2 | Amyloid-like protein 2 | Down in high Qp | 3.84 | Unchanged | Secretion |
| miR-30e-3p | Up in high Qp | ASPH* | Aspartyl/asparaginyl beta-hydroxylase (Fragment) | Down in high Qp | 2.11 | Unchanged |  |
| miR-30e-3p | Up in high Qp | ATP1A3 | Sodium/potassium-transporting ATPase subunit alpha | Down in high Qp | 1.57 | Unchanged |  |
| miR-30e-3p | Up in high Qp | ATP6V0D1 | V-type proton ATPase subunit | Down in high Qp | 1.61 | Unchanged | IRE1 mediated UPR |
| miR-30e-3p | Up in high Qp | ATP6V1A* | V-type proton ATPase catalytic subunit A | Down in high Qp | 1.51 | Unchanged |  |
| miR-30e-3p | Up in high Qp | CAMK2G* | Calcium/calmodulin-dependent protein kinase type II gamma chain | Down in high Qp | 1.52 | Unchanged | Secretion |
| miR-30e-3p | Up in high Qp | CANX* | Calnexin | Down in high Qp | 1.84 | Unchanged | Protein folding/ Secretion |
| miR-30e-3p | Up in high Qp | CHID1 | Chitinase domain-containing protein 1 | Down in high Qp | 2.33 | Unchanged | Secretion |
| miR-30e-3p | Up in high Qp | CROT* | Peroxisomal carnitine O-octanoyltransferase | Down in high Qp | 1.84 | Unchanged |  |
| miR-30e-3p | Up in high Qp | CS | Citrate synthase | Down in high Qp | 1.54 | Unchanged |  |
| miR-30e-3p | Up in high Qp | CTSB | Cathepsin B | Down in high Qp | 1.83 | Unchanged | Secretion |
| miR-30e-3p | Up in high Qp | ERLIN2* | Erlin-2 | Down in high Qp | 2.11 | Unchanged |  |
| miR-30e-3p | Up in high Qp | ERMP1* | Endoplasmic reticulum metallopeptidase 1 | Down in high Qp | 3.34 | Unchanged |  |
| miR-30e-3p | Up in high Qp | FAM114A1* | Protein Noxp20 (Fragment) | Down in high Qp | 1.85 | Unchanged |  |
| miR-30e-3p | Up in high Qp | FBXL20* | F-box/LRR-repeat protein 2 | Down in high Qp | 2.26 | Unchanged |  |
| miR-30e-3p | Up in high Qp | FDXR | NADPH:adrenodoxin oxidoreductase, mitochondrial | Down in high Qp | 1.61 | Unchanged |  |
| miR-30e-3p | Up in high Qp | FUCA1 | Tissue alpha-L-fucosidase | Down in high Qp | 2.55 | Unchanged | Secretion |
| miR-30e-3p | Up in high Qp | GALNS | N-acetylgalactosamine-6-sulfatase | Down in high Qp | 9.98 | Unchanged | Secretion |
| miR-30e-3p | Up in high Qp | GALNT2* | Polypeptide N-acetylgalactosaminyltransferase | Down in high Qp | 1.67 | Unchanged |  |
| miR-30e-3p | Up in high Qp | GANAB | Neutral alpha-glucosidase AB | Down in high Qp | 1.87 | Down in high Qp |  |
| miR-30e-3p | Up in high Qp | GCDH | Glutaryl-CoA dehydrogenase | Down in high Qp | 2.30 | Unchanged |  |
| miR-30e-3p | Up in high Qp | GLG1 | Golgi apparatus protein 1 | Down in high Qp | 2.39 | Unchanged |  |
| miR-30e-3p | Up in high Qp | GPD1 | Glycerol-3-phosphate dehydrogenase [NAD(+)] | Down in high Qp | 1.56 | Unchanged |  |
| miR-30e-3p | Up in high Qp | GRPEL1* | GrpE protein homolog | Down in high Qp | 1.75 | Unchanged | Protein folding |
| miR-30e-3p | Up in high Qp | HADH* | Hydroxyacyl-coenzyme A dehydrogenase, mitochondrial | Down in high Qp | 1.57 | Unchanged |  |
| miR-30e-3p | Up in high Qp | HEXA | Beta-hexosaminidase | Down in high Qp | 13.59 | Unchanged |  |
| miR-30e-3p | Up in high Qp | HYOU1 | Hypoxia up-regulated protein 1 | Down in high Qp | 2.07 | Unchanged | IRE1 mediated UPR |
| miR-30e-3p | Up in high Qp | IGF2R | Cation-independent mannose-6-phosphate receptor | Down in high Qp | 2.55 | Down in high Qp |  |
| miR-30e-3p | Up in high Qp | ITGAV* | Integrin alpha-V | Down in high Qp | 2.58 | Unchanged | Secretion |
| miR-30e-3p | Up in high Qp | KCNAB2 | Voltage-gated potassium channel subunit beta-2 | Down in high Qp | 1.82 | Unchanged | Secretion |
| miR-30e-3p | Up in high Qp | LAMB1* | Laminin subunit beta-1 | Down in high Qp | 1.92 | Unchanged |  |
| miR-30e-3p | Up in high Qp | LBR | Lamin-B receptor | Down in high Qp | 1.74 | Unchanged |  |
| miR-30e-3p | Up in high Qp | LMAN1 | Protein ERGIC-53 | Down in high Qp | 1.65 | Unchanged | Protein folding |
| miR-30e-3p | Up in high Qp | LRPAP1* | Alpha-2-macroglobulin receptor-associated protein | Down in high Qp | 1.77 | Unchanged |  |
| miR-30e-3p | Up in high Qp | MAN2A1* | Alpha-mannosidase 2 | Down in high Qp | 1.61 | Unchanged |  |
| miR-30e-3p | Up in high Qp | MAN2B1 | Lysosomal alpha-mannosidase | Down in high Qp | 2.37 | Unchanged | Secretion |
| miR-30e-3p | Up in high Qp | MLEC* | Malectin | Down in high Qp | 1.63 | Unchanged | Protein folding/ Secretion |
| miR-30e-3p | Up in high Qp | MYO6* | Myosin-VI | Down in high Qp | 2.01 | Unchanged |  |
| miR-30e-3p | Up in high Qp | NCEH1* | Arylacetamide deacetylase-like 1 | Down in high Qp | 2.36 | Unchanged |  |
| miR-30e-3p | Up in high Qp | NFS1 | Cysteine desulfurase, mitochondrial | Down in high Qp | 1.59 | Unchanged |  |
| miR-30e-3p | Up in high Qp | OCIAD1* | OCIA domain-containing protein 1 | Down in high Qp | 1.99 | Unchanged |  |
| miR-30e-3p | Up in high Qp | PDIA6 | Protein disulfide-isomerase A6 | Down in high Qp | 2.29 | Unchanged | Protein folding/ IRE1 mediated UPR |
| miR-30e-3p | Up in high Qp | PKLR | Pyruvate kinase | Down in high Qp | 1.67 | Unchanged |  |
| miR-30e-3p | Up in high Qp | PLD3 | Phospholipase D3 | Down in high Qp | 1.92 | Unchanged |  |
| miR-30e-3p | Up in high Qp | PLOD1* | Procollagen-lysine,2-oxoglutarate 5-dioxygenase 1 | Down in high Qp | 1.95 | Unchanged |  |
| miR-30e-3p | Up in high Qp | PLOD3* | Procollagen-lysine,2-oxoglutarate 5-dioxygenase 3 | Down in high Qp | 1.94 | Unchanged |  |
| miR-30e-3p | Up in high Qp | POFUT1 | GDP-fucose protein O-fucosyltransferase 2 | Down in high Qp | 3.56 | Down in high Qp |  |
| miR-30e-3p | Up in high Qp | PPWD1 | Peptidylprolyl isomerase | Down in high Qp | 2.39 | Unchanged |  |
| miR-30e-3p | Up in high Qp | PREB | Prolactin regulatory element-binding protein | Down in high Qp | 1.80 | Unchanged | Secretion/ IRE1 mediated UPR |
| miR-30e-3p | Up in high Qp | PROS1 | Vitamin K-dependent protein S | Down in high Qp | 11.46 | Unchanged | Secretion |
| miR-30e-3p | Up in high Qp | PTBP3 | Regulator of differentiation 1 | Down in high Qp | 1.83 | Unchanged |  |
| miR-30e-3p | Up in high Qp | PYGB* | Alpha-1,4 glucan phosphorylase | Down in high Qp | 1.55 | Unchanged | Secretion |
| miR-30e-3p | Up in high Qp | RAB11FIP3 | Rab11 family-interacting protein 3 | Down in high Qp | 2.86 | Unchanged |  |
| miR-30e-3p | Up in high Qp | RCN1* | Reticulocalbin-1 (Fragment) | Down in high Qp | 1.52 | Unchanged |  |
| miR-30e-3p | Up in high Qp | RPN1* | Dolichyl-diphosphooligosaccharide--protein glycosyltransferase subunit 1 | Down in high Qp | 1.80 | Unchanged |  |
| miR-30e-3p | Up in high Qp | SCP2 | Non-specific lipid-transfer protein | Down in high Qp | 1.67 | Unchanged |  |
| miR-30e-3p | Up in high Qp | SEL1L* | Protein sel-1-like 1 | Down in high Qp | 3.31 | Unchanged |  |
| miR-30e-3p | Up in high Qp | SEMA3E* | Semaphorin-3E | Down in high Qp | 77.41 | Unchanged |  |
| miR-30e-3p | Up in high Qp | SHMT2 | Serine hydroxymethyltransferase | Down in high Qp | 1.51 | Down in high Qp |  |
| miR-30e-3p | Up in high Qp | SLC25A12* | Calcium-binding mitochondrial carrier protein Aralar1 | Down in high Qp | 1.68 | Unchanged |  |
| miR-30e-3p | Up in high Qp | SLC25A13* | Calcium-binding mitochondrial carrier protein Aralar2 | Down in high Qp | 1.65 | Unchanged |  |
| miR-30e-3p | Up in high Qp | SLC25A20 | Mitochondrial carnitine/acylcarnitine carrier protein | Down in high Qp | 1.58 | Unchanged |  |
| miR-30e-3p | Up in high Qp | SLC27A2 | Long-chain-fatty-acid--CoA ligase 1 | Down in high Qp | 2.20 | Unchanged | Secretion |
| miR-30e-3p | Up in high Qp | SMC3* | Structural maintenance of chromosomes protein 3 | Down in high Qp | 1.51 | Unchanged |  |
| miR-30e-3p | Up in high Qp | SMPD1 | Sphingomyelin phosphodiesterase | Down in high Qp | 4.75 | Down in high Qp |  |
| miR-30e-3p | Up in high Qp | SOD1 | Superoxide dismutase | Down in high Qp | 2.42 | Unchanged | Secretion |
| miR-30e-3p | Up in high Qp | SRP72 | Signal recognition particle 72 kDa protein | Down in high Qp | 1.80 | Unchanged |  |
| miR-30e-3p | Up in high Qp | STAT3* | Signal transducer and activator of transcription | Down in high Qp | 1.65 | Unchanged |  |
| miR-30e-3p | Up in high Qp | STIM1 | Stromal interaction molecule 1 | Down in high Qp | 1.70 | Unchanged |  |
| miR-30e-3p | Up in high Qp | STT3B | Dolichyl-diphosphooligosaccharide--protein glycosyltransferase subunit STT3B | Down in high Qp | 1.76 | Unchanged |  |
| miR-30e-3p | Up in high Qp | SYNJ2BP | Synaptojanin-2-binding protein | Down in high Qp | 1.81 | Unchanged |  |
| miR-30e-3p | Up in high Qp | TGM2* | Protein-glutamine gamma-glutamyltransferase 2 | Down in high Qp | 3.05 | Unchanged |  |
| miR-30e-3p | Up in high Qp | TIMM9 | Mitochondrial import inner membrane translocase subunit Tim9 | Down in high Qp | 1.77 | Unchanged |  |
| miR-30e-3p | Up in high Qp | TMED4 | Transmembrane emp24 domain-containing protein 4 | Down in high Qp | 1.97 | Unchanged |  |
| miR-30e-3p | Up in high Qp | TMED7* | Transmembrane emp24 domain-containing protein 7 | Down in high Qp | 1.65 | Unchanged |  |
| miR-30e-3p | Up in high Qp | TMEM43* | Transmembrane protein 43 | Down in high Qp | 1.75 | Unchanged |  |
| miR-30e-3p | Up in high Qp | TMTC3* | Transmembrane and TPR repeat-containing protein 3 | Down in high Qp | 3.35 | Unchanged |  |
| miR-30e-3p | Up in high Qp | TPM1 | Tropomyosin alpha-1 chain | Down in high Qp | 2.47 | Unchanged |  |
| miR-30e-3p | Up in high Qp | TPP1 | Tripeptidyl-peptidase 1 | Down in high Qp | 2.17 | Unchanged | IRE1 mediated UPR |
| miR-30e-3p | Up in high Qp | UGGT1 | UDP-glucose:glycoprotein glucosyltransferase 1 | Down in high Qp | 2.30 | Down in high Qp |  |
| miR-30e-3p | Up in high Qp | YARS* | Tyrosine--tRNA ligase | Down in high Qp | 1.55 | Unchanged |  |

Supplementary Table 5: Predicted targets of miR-30e-3p which were shown to be down in high Qp at protein level at day 6. (* indicates the protein was identified as a predicted target of the miRNA by >3 databases).

| DE protein | Protein description | Protein Phenotype | Fold change | mRNA Phenotype | Predicted target of: |
| --- | --- | --- | --- | --- | --- |
| ACAD11 | Acyl-CoA dehydrogenase family member 11 | Down in high Qp | 1.92 | Unchanged | miR-200a, miR-30e, miR-878 |
| CANX | Calnexin | Down in high Qp | 1.84 | Unchanged | miR-200a, miR-30e, miR-878 |
| PPWD1 | Peptidylprolyl isomerase | Down in high Qp | 2.39 | Unchanged | miR-200a, miR-30e, miR-878 |
| SEMA3 | Semaphorin-3E | Down in high Qp | 77.41 | Unchanged | miR-200a, miR-30e, miR-878 |
| ACBD5 | Acyl-CoA-binding domain-containing protein 5 | Down in high Qp | 1.80 | Unchanged | miR-30e, miR-878 |
| ERLIN2 | Erlin-2 | Down in high Qp | 2.11 | Unchanged | miR-30e, miR-878 |
| SEL1L | Protein sel-1-like 1 | Down in high Qp | 3.31 | Unchanged | miR-30e, miR-878 |
| STT3B | Dolichyl-diphosphooligosaccharide--protein glycosyltransferase subunit STT3B | Down in high Qp | 1.76 | Unchanged | miR-30e, miR-878 |
| ABCC8 | ATP-binding cassette sub-family D member 3 (Fragment) | Down in high Qp | 2.09 | Unchanged | miR-200a, miR-30e, |
| ACTA2 | Actin, aortic smooth muscle | Down in high Qp | 2.05 | Unchanged | miR-200a, miR-30e, |
| APLP2 | Amyloid-like protein 2 | Down in high Qp | 3.84 | Unchanged | miR-200a, miR-30e, |
| ASPH | Aspartyl/asparaginyl beta-hydroxylase (Fragment) | Down in high Qp | 2.11 | Unchanged | miR-200a, miR-30e, |
| ATP6V0S1 | V-type proton ATPase subunit | Down in high Qp | 1.61 | Unchanged | miR-200a, miR-30e, |
| ATP6V1A | V-type proton ATPase catalytic subunit A | Down in high Qp | 1.51 | Unchanged | miR-200a, miR-30e, |
| CAMK2G | Calcium/calmodulin-dependent protein kinase type II gamma chain | Down in high Qp | 1.52 | Unchanged | miR-200a, miR-30e, |
| CHID1 | Chitinase domain-containing protein 1 | Down in high Qp | 2.33 | Unchanged | miR-200a, miR-30e, |
| CTSB | Cathepsin B | Down in high Qp | 1.83 | Unchanged | miR-200a, miR-30e, |
| ERMP1 | Endoplasmic reticulum metallopeptidase 1 | Down in high Qp | 3.34 | Unchanged | miR-200a, miR-30e, |
| FAM114A1 | Protein Noxp20 (Fragment) | Down in high Qp | 1.85 | Unchanged | miR-200a, miR-30e, |
| FBXL20 | F-box/LRR-repeat protein 2 | Down in high Qp | 2.26 | Unchanged | miR-200a, miR-30e, |
| FUCA1 | Tissue alpha-L-fucosidase | Down in high Qp | 2.55 | Unchanged | miR-200a, miR-30e, |
| GALNT2 | Polypeptide N-acetylgalactosaminyltransferase | Down in high Qp | 1.67 | Unchanged | miR-200a, miR-30e, |
| GPD1 | Glycerol-3-phosphate dehydrogenase [NAD(+)] | Down in high Qp | 1.56 | Unchanged | miR-200a, miR-30e, |
| HYOU1 | Hypoxia up-regulated protein 1 | Down in high Qp | 2.07 | Unchanged | miR-200a, miR-30e, |
| ITGAV | Integrin alpha-V | Down in high Qp | 2.58 | Unchanged | miR-200a, miR-30e, |
| KCNAB2 | Voltage-gated potassium channel subunit beta-2 | Down in high Qp | 1.82 | Unchanged | miR-200a, miR-30e, |
| LAMB1 | Laminin subunit beta-1 | Down in high Qp | 1.92 | Unchanged | miR-200a, miR-30e, |
| LBR | Lamin-B receptor | Down in high Qp | 1.74 | Unchanged | miR-200a, miR-30e, |
| MAN2A1 | Alpha-mannosidase 2 | Down in high Qp | 1.61 | Unchanged | miR-200a, miR-30e, |
| PLD3 | Phospholipase D3 | Down in high Qp | 1.92 | Unchanged | miR-200a, miR-30e, |
| RPN1 | Dolichyl-diphosphooligosaccharide--protein glycosyltransferase subunit 1 | Down in high Qp | 1.80 | Unchanged | miR-200a, miR-30e, |
| SMC3 | Structural maintenance of chromosomes protein 3 | Down in high Qp | 1.51 | Unchanged | miR-200a, miR-30e, |
| SRP72 | Signal recognition particle 72 kDa protein | Down in high Qp | 1.80 | Unchanged | miR-200a, miR-30e, |
| STAT3 | Signal transducer and activator of transcription | Down in high Qp | 1.65 | Unchanged | miR-200a, miR-30e, |
| SYNJ2BP | Synaptojanin-2-binding protein | Down in high Qp | 1.81 | Unchanged | miR-200a, miR-30e, |

Supplementary Table 6: Overlapping targets between miR-200a, miR-30e and miR-878.

A

B
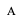


C
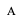


D
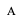


Supplementary Figure 1 (A) Viability (B) Viable cell density (C) Titer and (D) Qp of a CHO cell CDCL transfected with a negative control mimic, miR-30e-3p mimic and a miR-878 mimic. Cell free supernatant was analysed by HPLC and UvVis absorbance values were obtained. A standard curve for the known product was used to determine titer and Qp.

Supplementary Figure 2: Relative abundance of miRNAs in negative control and pre-miR treated cells. Relative abundance is displayed in ∆Ct values. Higher values indicate a lower abundance. Error bars represent standard deviation between high or low Qp CDCLs.

Supplementary Figure 3: Relative quantification (RQ) of identified miRNAs in pri-miR treated and Negative Control pri-miR treated cells. Relative miRNA abundance was determined by qRT-PCR using the ddCt method with U6 snRNA as an endogenous control.
